# Insulin use promotes pro-inflammatory changes in the transcriptome of atherosclerotic plaques in patients with diabetes mellitus^☆^

**DOI:** 10.1016/j.jmccpl.2025.100829

**Published:** 2025-11-16

**Authors:** Angelle Bradford, Tadashi Yoshida, Sergiy Sukhanov, Foster F. Woods, Patrice Delafontaine, Hernan A. Bazan, T. Cooper Woods

**Affiliations:** a Departments of Physiology and Medicine, Tulane University School of Medicine, New Orleans, LA, USA; b Section of Vascular/Endovascular Surgery, Department of Surgery, Ochsner Clinic Foundation, New Orleans, LA, USA; c The University of Queensland Faculty of Medicine, Ochsner Clinical School, New Orleans, LA, USA

**Keywords:** Plaque rupture, Diabetes mellitus, Atherosclerosis, Stroke, Transcriptome

## Abstract

**Background:**

Diabetes Mellitus is associated with increased risk of myocardial infarctions and strokes due to accelerated atherosclerotic plaque development and rupture. The mechanisms driving plaque rupture in the diabetic setting remain unclear.

**Methods:**

We sequenced ribosome-depleted total RNA from carotid plaques obtained from diabetic subjects undergoing carotid endarterectomy with high-grade stenosis, who either recently experienced a carotid-related ischemic cerebrovascular event (Symptomatic, *n* = 6) or had no such event (Asymptomatic, n = 6).

**Results:**

Principal component analysis of the most variable transcripts demonstrated that sex and insulin use, rather than plaque rupture status, were the major contributors to the variability in the dataset. Hierarchical clustering further highlighted the importance of these factors in the dataset but also included two clusters whose expression was elevated in the symptomatic group. These clusters were enriched with transcripts for immunoglobulins and matrix metalloproteinases, suggesting increased B-cell infiltration and extracellular matrix degradation in the symptomatic plaques. We identified only two transcripts, *RNU2–1* and *PI4KAP1*, as significantly up-regulated in the symptomatic group. We identified 35 transcripts with altered expression in subjects on insulin therapy, including regulators of B-cells (*MZB1*), T cells (*BTN3A1, BTN3A2, BTN3A3, CD96*, and *CTSW*), and macrophages (*MPEG1*). Next, we used spatial deconvolution to focus on differentially expressed genes in the fibrous cap of the carotid plaques. In the symptomatic group, we identified elevated levels of five angiogenesis-associated transcripts (*ALS2CL, EPHB4, SMAD1, PREX2, EFNA5*), suggesting increased intraplaque neovascularization in ruptured plaques.

**Conclusions:**

Thus, insulin use significantly impacts the transcriptome of atherosclerotic plaques, promoting vascular inflammation.

## Highlights

• In diabetes, insulin use is a primary contributor to transcriptome variability and promotes vascular inflammation.
• Clustering groups transcripts for matrix metalloproteinases and immunoglobins in a similar manner as in non-diabetics.
• Spatial deconvolution shows the fibrous cap of ruptured plaques in diabetics is enriched for angiogenic transcripts.

## Graphical abstract

**Figure:**
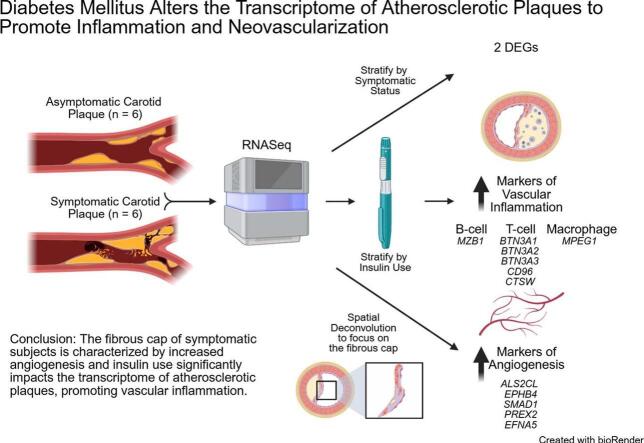

## 1 Introduction

The diabetic population is at a 2-to-4-fold increased risk for heart attack and stroke due to an accelerated development of atherosclerotic plaques. The rupture of atherosclerotic plaques releases thromboemboli into the blood, leading to ischemic events, including myocardial infarctions and strokes. [1,2] Given the disproportionately high prevalence of cerebrovascular events in diabetic individuals, understanding the changes in the plaque transcriptome specific to this population is essential for developing targeted interventions to reduce morbidity and mortality associated with diabetes mellitus.

Stable atherosclerotic plaques are characterized by a protective fibrous cap overlying the lipid-rich necrotic core of the plaque. [3] The fibrous cap is formed by vascular smooth muscle cells (VSMCs) that migrate from the media to the intima of the artery, proliferate, and secrete collagen. [4] Thinning of the fibrous cap in the late stages of atherosclerosis creates a rupture-prone thin cap fibroatheroma. [5] Ruptured plaques have thinner overlying fibrous caps with reduced VSMC and collagen content. Macrophage, T-cell, B-cell infiltration, and neovascularization also characterize the ruptured plaque. [2,6] While these factors have been implicated in atherosclerotic plaque development in diabetes, further data is needed to understand their role in plaque rupture. [7,8]

Type 2 diabetes mellitus, a chronic metabolic disorder, arises from the development of peripheral insulin resistance coupled with inadequate insulin secretion from the pancreatic β-cells to maintain normal glucose homeostasis. Though treatment begins with lifestyle changes and progresses to the use of one or more oral anti-hyperglycemic agents, ultimately many patients will require insulin therapy. [9] The Look AHEAD trial found that of the 3913 type 2 diabetic patients who were not on insulin therapy at baseline, 27.8 % initiated insulin therapy within the subsequent ten years. [10] A more recent study in 24,360 patients treated with metformin found that 10 % began insulin therapy within 5 years. [11] As insulin has direct effects on the vasculature, the role of insulin therapy in the cardiovascular complications of diabetes warrants consideration.

We previously characterized the transcriptome of recently ruptured carotid plaques in non-diabetic subjects, highlighting their enrichment in transcripts linked to B-cell function and matrix metalloproteinases. [12] Here, we identify insulin and sex as the major contributors to differences in the transcriptomes of asymptomatic and recently (~ 5 days) ruptured carotid plaques of diabetic subjects, including using spatial deconvolution to identify active mechanisms that operate in the fibrous cap.

## 2 Materials and methods

### 2.1 Sample collection

Carotid plaque specimens and relevant medical histories were acquired from a total of 12 patients with a prior diagnosis of type 2 diabetes undergoing carotid endarterectomy (CEA) in the Section of Vascular/Endovascular Surgery in the Department of Surgery at the Ochsner Clinic in New Orleans, LA. Samples were stratified according to their sex, whether they were on insulin therapy (Insulin Use), and whether they had a recent ischemic cerebrovascular event (Status). Those without a recent ischemic cerebrovascular event (asymptomatic) included patients undergoing CEA without a previous neurologic event but with high-grade carotid stenosis (*n* = 6). The symptomatic group (n = 6) consists of patients undergoing CEA within an average of 5 days of an acute neurologic event (transient ischemic attack or acute stroke). The samples were immediately snap-frozen and stored in liquid nitrogen until processing for RNA isolation.

All procedures in this study were performed following the ethical standards of the 1964 Helsinki Declaration. This study was approved by the Ochsner Health Institutional Review Board (IRB protocol # 2010.130B). All participants gave written informed consent for their participation in the study.

### 2.2 RNA isolation

Total RNA was obtained from the carotid plaques as previously described. [12] The plaque tissue was sectioned into six transverse segments of ~1 cm in length, and plaque content was determined via macroscopic examination. Sections containing complicated lesions were homogenized in TRIzol Reagent (ThermoFisher) using a rotor-stator homogenizer, and total RNA was isolated using the miRNeasy Mini Kit (Qiagen).

### 2.3 RNA-sequencing

Library preparation and sequencing were performed by The University Wisconsin Biotechnology Center. Ribosomal RNA was reduced using the RiboZero assay (EpiCentre-Illumina), and an RNA-Seq library was constructed with the stranded TruSeq RNA kit (Illumina). Quality of the RNA sample and DNA library was confirmed using the 2100 Bioanalyzer system. Using a HiSeq2000 (Illumina), 1 × 100 bp sequencing was performed. Sequence alignment to the human genome (hg19 assembly) was performed by the Tulane Next Generation Sequence Analysis Core using Novoalign (Novocraft), and splice junctions were identified using TopHat. [13] Coverage files and FPKM values were generated using SAMMate, and coverage was visualized on the Integrated Genome Viewer. [14,15] The sequencing data generated were deposited in the Gene Expression Omnibus (GEO) under accession number GSE311535.

### 2.4 Transcriptome analysis

Transcript abundance estimates were imported using *tximport* package and principal component analysis (PCA), hierarchical clustering, and identification of differentially expressed genes (DEGs) were performed in an unsupervised manner using the *DESeq2* package. [16,17] DEGs were defined as those transcripts with a p-adjusted value <0.05 with a fold change >2. Spatial deconvolution was performed using the *bulk2space* package. [18] The GSE260657 dataset was used as the single-cell reference and GSE220218 was used as the spatial reference. [19,20] For *bulk2space*, the default parameters were used.

### 2.5 Statistical analysis

All analyses were performed using R 4.4.0 and Python 3.8. Wilcoxon rank-sum test and Pearson correlation coefficient were used to determine differential gene expression and genetic similarity. *P* < 0.05 was considered statistically significant. Bonferroni correction was used for multiple testing.

## 3 Results

### 3.1 Patient population characteristics

Carotid plaques were obtained from 12 patients undergoing carotid endarterectomy (CEA) with a diagnosis of diabetes. All patients had significant carotid plaques, with six being asymptomatic and six having had a recent ischemic cerebrovascular event (Table 1). There was no significant difference in age, body mass index, lipid profile, serum creatinine, estimated glomerular filtration rates, percent stenosis, or hemoglobin A1c between the two patient groups. Additionally, there were no significant differences in the rates of smoking, alcohol use, or chronic kidney disease between the groups. Each group had similar insulin, metformin, aspirin, statin, and clopidogrel use. The time between the cerebrovascular event and the CEA in the symptomatic group was 5.3 ± 0.8 days.

**Table 1:** Patient Characteristics.

	Total			Asymptomatic			Symptomatic			
Characteristics	(*n* = 12)			(*n* = 6)			(n = 6)			*P*-value
Age, y	70.9	±	2.5	68.8	±	4.5	73	±	2.2	0.43
Sex, % Male	6		(50 %)	6		(50 %)	6		(50 %)	1.00
Body Mass Index, kg/m^2^	24.5	±	4.3	29.6	±	1.9	20.0	±	6.5	0.20
History of Smoking	7		(58 %)	4		(67 %)	3		(50 %)	0.56
History of Ethanol Use	3		(25 %)	1		(17 %)	2		(33 %)	0.51
Total Cholesterol, mg/dL	161.8	±	14.6	165.2	±	16.0	157.5	±	29.0	0.83
HDL, mg/dL	38.2	±	2.2	40.4	±	3.5	36.0	±	2.5	0.34
LDL, mg/dL	86.2	±	11.4	79.92	±	7.2	94.0	±	25.7	0.63
Triglycerides, mg/dL	203.7	±	35.5	213.8	±	41.9	193.6	±	62.0	0.80
Serum Creatinine, mg/dL	1.1	±	0.1	1.1	±	0.1	1.08333	±	0.1	1.00
eGFR, mL/min/1.73m^2^	74.9	±	4.9	74.8	±	6.6	74.9	±	7.8	0.12
Chronic Kidney Disease	4		(33 %)	2		(33 %)	2		(33 %)	1.00
Percent Stenosis	82.3	±	3.4	82.0	±	3.5	82.5	±	6.1	0.29
Hemoglobin A1c	8.0	±	0.5	8.1	±	0.8	7.9	±	0.6	0.86
Insulin Use	6		(50 %)	3		(50 %)	3		(50 %)	1.00
Metformin Use	7		(58 %)	3		(50 %)	4		(67 %)	0.56
Aspirin Use	10		(83 %)	4		(67 %)	6		(100 %)	0.12
Statin Use	11		(92 %)	5		(83 %)	6		(100 %)	0.30
Clopidogrel Use	5		(42 %)	3		(50 %)	2		(33 %)	0.56

Values are means ± SE; HDL, High Density Lipoprotein. LDL, Low Density Lipoprotein. eGFR, estimated Glomerular Filtration Rate.

### 3.2 Principal component analysis of highly variable transcripts suggests sex and insulin use are the major contributors to the variability in the transcriptome

**Fig. 1:**
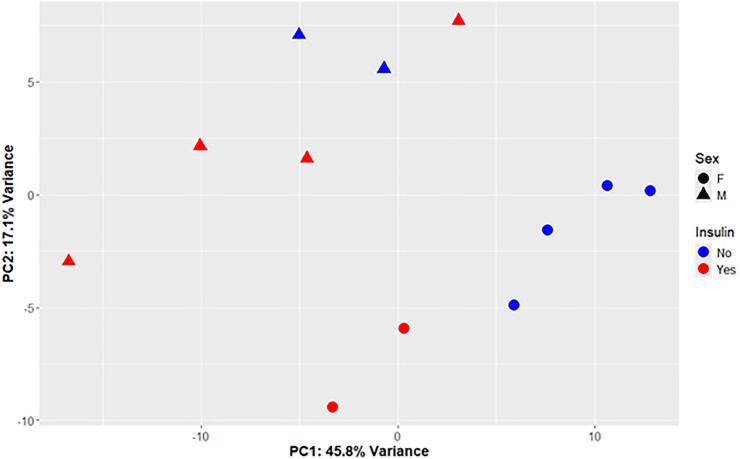
Principal component analysis (PCA) indicates sex and insulin use are associated with alterations in the transcriptome. PCA plot of the top 57 most variable transcripts in asymptomatic and recently ruptured plaques. Triangles (▲) indicate male and circles (●) indicate female subjects. Subjects on insulin therapy are colored red and those that are not on insulin are blue. (For interpretation of the references to colour in this figure legend, the reader is referred to the web version of this article.)

We performed RNA-Seq on ribosome-depleted total RNA obtained from the carotid plaques to compare the transcriptome of plaques before and after rupture. PCA of the 57 most variable transcripts achieved the greatest separation along the first principal component (PC1), which accounts for only 45.8 % of the total variability (Fig. 1). The separation included overlapping asymptomatic and symptomatic samples and adding additional transcripts did not enhance the separation. Additionally, the separation did not relate to hemoglobin A1c or metformin use. The samples were clustered according to sex into two groups separated along the diagonal. Additionally, there was partial separation according to insulin use along PC1. This contrasts with our previous study, which had similar samples from non-diabetic male subjects, where a recent stroke was the dominant factor underlying variability in the transcriptome. [12] This suggests the presence of diabetes significantly impacts the transcriptome of arteries.

### 3.3 Hierarchical clustering of highly variable transcripts identifies clusters associated with plaque rupture in addition to sex and insulin use

**Fig. 2:**
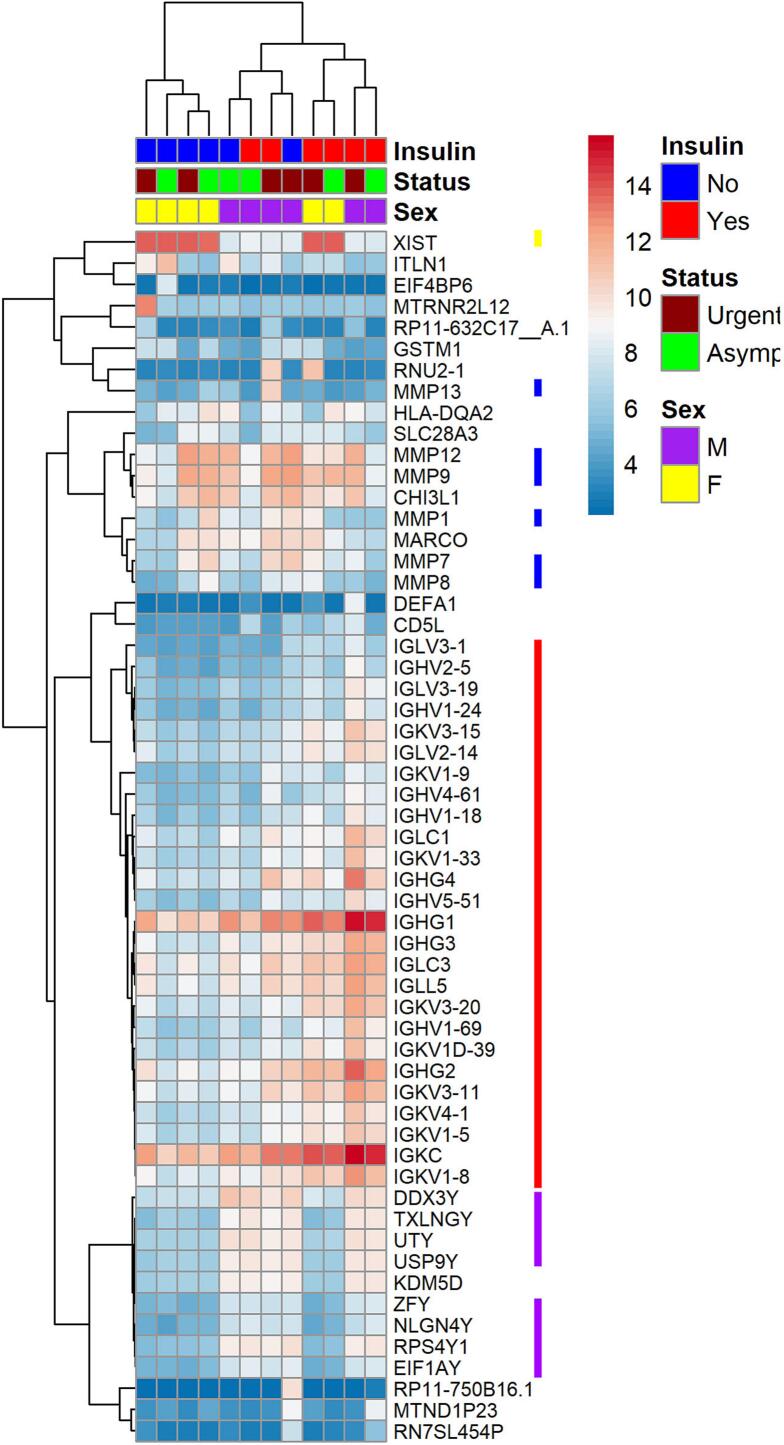
Hierarchical clustering identifies groups of co-expressed transcripts in atherosclerotic plaques. Clustering of the top 57 transcripts in carotid plaques differentiates between asymptomatic and recently ruptured plaques. Bars adjacent to the cluster indicate locations of transcripts associated with female sex (yellow) male sex (green), metalloproteinases (blue), and immunoglobulins (red).

To further examine the changes in gene expression that occur with plaque rupture in the diabetic population, we performed hierarchical clustering of the transcripts in the carotid plaques (Fig. 2). No set of transcripts led to clustering that separated the ruptured and asymptomatic samples. Clustering of the 57 most variable transcripts partially grouped the samples from patients by sex and whether they use insulin. These groups overlap, making it difficult to differentiate the relationship between sex or insulin use and the transcripts' expression. Two clusters appeared to coincide with sex. A cluster that included an X chromosome-related transcript, *XIST,* exhibited elevated expression in females. Similarly, a cluster with elevated expression in males contained several Y-linked transcripts (*DDX3Y, TXLNGY, UTY, USP9Y, ZFY, NLGH4Y*). An additional cluster included multiple matrix metalloproteinases (e.g., *MMP1, MMP7, MMP8, MMP9, MMP12)* and another included immunoglobulins. This clusters exhibited partial alignment with plaque rupture status and showed increased expression in the symptomatic group, similar to our previous study in non-diabetic subjects. Thus, while systemic factors such as sex and insulin use are the primary drivers of variability in the transcriptome, the transcriptome of the plaque is altered by plaque rupture.

### 3.4 Differential gene expression associated with insulin use suggests increased vascular inflammation

We next looked at DEGs associated with plaque rupture in diabetic patients. Only two transcripts were identified: a small nuclear RNA (*RNU2–1)* and a pseudogene (*PI4KAP1).* Given that rupture status did not appear as a major contributor to the variability between samples, the fact that very few DEGs related to plaque rupture is not surprising. As our clustering analysis suggested insulin use as a contributor to the variability of the dataset, we examined the DEGs associated with a subject's insulin use (Table 2). The majority of the transcripts elevated in the insulin use group are related to immune system functions, including several encoding immunoglobulins and regulators of the function of B cells (*MZB1),* T cells (*BTN3A1, BTN3A2, BTN3A3, CD96,* and *CTSW*), and macrophages (*MPEG1*). *BTN3A1* and *BTN3A3*, upregulated in insulin users, are critical in modulating T-cell function, suggesting that insulin therapy may enhance adaptive immune activity within plaques. This aligns with the observed increased expression of *MPEG1*, a macrophage activation marker, pointing to amplified vascular inflammation in diabetic plaques. There were also increases in two transcripts associated with vascular smooth muscle cell proliferation (*UTS2* and *ADAM22*). These results suggest that insulin-dependent diabetes may be related to increased inflammation in atherosclerotic plaques.

**Table 2:** Differentially Expressed Genes Associated with Insulin Use.

Gene Symbol	Mean Expression	Log_2_ (Fold Change)	Standard Error	Adjusted P-value
RNU2–1	260	25.48	3.00	< 0.01
IGHV6–1	24	7.40	1.49	< 0.01
IGKV3–20	1478	4.10	0.92	0.02
IGKV1–17	95	4.04	0.91	0.02
IGLV2–18	33	3.72	0.71	< 0.01
IGHV3–33	289	3.61	0.75	< 0.01
IGHV1OR15–1	31	3.45	0.78	0.02
IGHV4–55	32	3.42	0.83	0.04
IGHV3–49	116	3.41	0.85	0.05
IGHV3–73	47	3.31	0.75	0.02
UTS2	16	2.91	0.64	0.01
MZB1	130	2.82	0.64	0.02
IL5RA	23	1.95	0.45	0.02
TDRD15	17	1.93	0.47	0.04
LINC00612	32	1.79	0.44	0.04
C1orf200	25	1.26	0.31	0.04
MPEG1	3406	1.15	0.27	0.02
PYHIN1	145	1.10	0.27	0.05
C5orf63	69	1.06	0.24	0.02
GPR126	253	1.02	0.25	0.04
SVEP1	1099	0.99	0.23	0.02
GIMAP1	207	0.94	0.18	< 0.01
BTN3A2	840	0.90	0.20	0.02
ADAM22	131	0.85	0.19	0.01
IQCH-AS1	116	0.85	0.18	0.01
CD96	205	0.83	0.21	0.05
CTSW	89	0.78	0.17	0.01
BTN3A3	629	0.69	0.17	0.05
BTN3A1	921	0.64	0.15	0.03
PCBP1-AS1	811	0.25	0.06	0.04
AFF4	4208	−0.28	0.07	0.05
TP53BP2	1211	−0.44	0.11	0.03
TCTN2	268	−0.46	0.11	0.04
SPSB1	567	−0.88	0.21	0.04
MURC	36	−0.98	0.20	< 0.01

### 3.5 The fibrous cap of symptomatic plaques is enriched with transcripts associated with neovascularization

While the effects of diabetes are likely consistent throughout the artery, the transcriptome of the fibrous cap is the most important in determining plaque vulnerability. Thus, comparing the transcripts differentially expressed in the fibrous cap versus the media of carotid plaques may reveal transcripts associated with plaque rupture in diabetic subjects. We used spatial deconvolution to resolve the carotid plaque transcriptome. We compared the difference in fold change of transcripts in the fibrous cap versus media between the ruptured and asymptomatic subjects (Table 3). All but one of the DEGs elevated in the symptomatic group (*ALS2CL, EPHB4, SMAD1, PREX2, EFNA5*) are associated with plaque neovascularization, a process commonly found in vulnerable plaques (Table 3). [6] There were five transcripts with lower expression in the fibrous cap of symptomatic plaques, but these did not exhibit a common function in the vasculature. The upregulation of angiogenesis-related genes such as *SMAD1* and *PREX2* in symptomatic plaques suggests a key role for intraplaque neovascularization in the development of plaque instability.

**Table 3:** Differentially Expressed Genes in the Fibrous Cap Associated with Plaque Rupture.

	Mean Log_2_ (Fold Change)					
Gene Symbol	Symptomatic	Asymptomatic	Delta	Standard error	p-value	Putative Function
ALS2CL	0.34	−0.45	0.79	0.15	0.05	Regulation of Rab5 /Angiogenesis
EPHB4	0.30	−0.48	0.79	0.17	0.02	Angiogenesis
SMAD1	0.25	−0.49	0.74	0.14	0.01	Angiogenesis
FXYD6-FXYD2	0.22	−0.50	0.72	0.12	0.02	
PREX2	0.04	−0.66	0.70	0.15	0.04	Angiogenesis
EFNA5	−0.17	−0.47	0.30	0.04	0.01	Angiogenesis
NEK3	−0.21	0.14	−0.36	0.06	0.05	Cell proliferation
SLC8A1	0.08	0.44	−0.36	0.09	0.03	Ion channel / VSMC proliferation
ZKSCAN5	−0.23	0.13	−0.36	0.07	0.02	Transcription
RASEF	−0.66	0.17	−0.83	0.19	0.04	Inhibition of vascular remodeling
LIG4	−0.55	0.28	−0.83	0.12	0.02	DNA Repair

## 4 Discussion

Previously, we performed a study in non-diabetic male subjects and found clear differences between those subjects with a recent cerebrovascular event and those without. The current study was designed to identify differences in the transcriptome associated with plaque rupture in diabetes. The data instead show that in the setting of diabetes, numerous factors contribute to the variability of the transcriptome, complicating the identification of the transcripts associated with plaque rupture.

That aspects of diabetes would promote changes in the carotid transcriptome is not unexpected. Singh et al. found lower expression of transforming-growth factor β in carotid plaques of type 2 diabetic patients. [21] We have reported the elevation of microRNA-221 and -222 in both non-diseased and diseased arteries of diabetic subjects. [22,23] Stratification of the samples according to insulin use identified several transcripts suggesting increased vascular inflammation in subjects on insulin therapy. On the other hand, Narayanan et al. did not observe differences between the cellular composition of carotid plaques from diabetic versus non-diabetic patients using deconvolution of bulk microarray data. [24] This study included samples from up to six months after the ischemic cerebrovascular event, potentially accounting for this difference with our study. Focusing on a specific subset of diabetic subjects, for example, only insulin-dependent subjects, might minimize the variability induced by diabetes and allow for a better examination of the mechanisms leading to plaque rupture.

We examined the ability of PCA to separate the samples according to their clinical scenarios. We found that there was no set of transcripts that completely separated the samples along a single principal component. The best clustering was achieved with a relatively small number of transcripts and accounted for only 45.8 % of the variance of the dataset. The PCA suggests that sex and insulin use were the most substantial contributors to variability between samples and not plaque rupture as we observed previously. The hierarchical clustering exhibited a similar lack of grouping according to clinical characteristics. Examining the individual clusters reveals that one was enriched for matrix metalloproteases and another for immunoglobulins. These clusters, which exhibit elevated levels in the symptomatic group, were also observed in our previous study in non-diabetic subjects. [12] In fact, the clusters are almost identical in the two studies with only two additional transcripts being included in the previous study. Thus, in presence of diabetes there are processes, namely B cell infiltration and extracellular matrix degradation that occur in a similar manner in unstable plaques.

We identified several DEGs that were associated with insulin use. Binding of insulin to its receptor results in the activation of the Akt and Extracellular signal Response Kinase 1/2 (ERK1/2) pathways. We and others have found increased ERK1/2 activity compared to Akt activity in diabetic models. [25,26] Several of the up-regulated DEGs are known to be increased in response to ERK1/2 activation or its downstream mediators. An inflammatory gene, *IL5RA*, is increased in myeloid cells where ERK1/2 activity has been stimulated by triciribine. [27] *SVEP1* is a gene associated with increased vascular smooth muscle cell proliferation and migration in atherosclerotic plaque development and its expression is induced by EGR-1, an early response gene downstream of ERK1/2. [28] Urotensin II, which is encoded by *UTS2*, is a potent vasoconstrictor that contributes to atherosclerosis. [29] Urotensin II expression is increased in response to Activator Protein-1, a transcription factor downstream of the ERK1/2 pathway. [30] *ADAM22*, is associated with increased plaque development and is regulated by the steroid coactivator, SRC-1, which also participates in co-activation of AP-1. [[31], [32], [33]] Thus, it is possible that the up-regulated DEGs associated with insulin use are resulting from increased insulin activation of the ERK1/2 pathway and promote vascular inflammation.

Several other studies have found that members of the matrix metalloproteinase enriched cluster (*MMP1, MMP8, MMP9,* and *MMP12*) were also found to up-regulated in unstable carotid plaques obtained from patients undergoing CEA. [[34], [35], [36], [37]] MMP1 and MMP7 were elevated in the serum of patients with unstable versus carotid plaques. [38] The matrix metalloproteinases found in this cluster degrade diverse components of the fibrous cap, including collagen (*MMP1* and *MMP8*), fibronectin (*MMP7*), gelatin (*MMP9*), and elastin (*MMP12*). [39,40] Elevated MMP1 and MMP9 were also correlated with an increase in plaque angiogenesis and vulnerability in a rabbit model of atherosclerosis. [41,42]

Our previous study also found increased immunoglobulins and other transcripts associated with B cell function. [12] As stated above, the immunoglobulins identified as increased in the symptomatic plaques were also observed clustered together in our previous study. While macrophages are the most common inflammatory cells found in the fibrous cap, B cells are present in the vulnerable sections of fibrous caps of carotid plaques. [2] The increased immunoglobulins do not include IgMs, suggesting this may represent an increase in follicular B cells. This subset of B cells is proatherogenic and promotes plaque vulnerability. [43,44] Thus, while plaque rupture may not be the dominant factor regulating gene expression in carotid plaques under diabetic conditions, the hierarchical clustering suggests some of the same mechanisms seen in the non-diabetic setting appear to be active.

Intraplaque neovascularization is associated with atherosclerotic plaque vulnerability and is increased in diabetic patients. [6,7] Here, we used spatial deconvolution to identify transcripts that increased in the fibrous cap of the symptomatic subjects. All but one of the transcripts identified have putative roles in intraplaque neovascularization and angiogenesis (Table 3). ALS2CL and its homolog, ALS2, are involved in the regulation of Rab5 activity, which plays a role in angiogenesis. [45] Rab5 activity in atherosclerotic plaques has also been implicated in the loss of VSMCs in vulnerable plaques. [46] *EPHB4* and *EFNA5* encode members of the Eph family of receptors and ligands that have roles in angiogenesis. [47,48] *PREX2* is a Rho guanine nucleotide exchange factor that promotes endothelial cell migration. [49] *SMAD1* deficiency is embryonically lethal due to impaired vessel development. [50] Together, the increase in these transcripts suggests intraplaque neovascularization may be increased in the ruptured plaques of diabetic subjects.

A strength of the current study is the timing of the sample collection. The samples were collected on average 5.3 days after the ischemic cerebrovascular event. We have previously reported that two microRNAs are down-regulated following plaque rupture with their expression returning to normal within 7 days. [22] As CEAs are more commonly performed months after an ischemic cerebrovascular event, our carotid samples gives us a more accurate picture of the transcriptome during rupture. Nonetheless, we recognize that some of the identified changes are a result rather than a cause of the rupture. A key limitation of the design of this study was the lack of inclusion of non-diabetic subjects in the current data collection. Our hope was to directly compare with our previously published non-diabetic dataset as it was collected at the same time and processed in a similar matter. Unfortunately, we found that batch effects prevented this approach. An additional limitation of this study is the reliance on bulk RNA-seq, which lacks single-cell resolution. However, the use of spatial deconvolution circumvents this by providing regional insights, such as identifying fibrous cap-specific expression patterns critical to plaque rupture. Still, our data highlights the need to use spatial transcriptomic methods to examine the changing transcriptome in vulnerable plaque. Finally, given the variability of data, future studies that control for differences in diet may better identify the differences between asymptomatic and rupture plaque transcriptomes.

The primary finding of this report is that the presence of diabetes promotes changes in the transcriptome that promote vascular inflammation. These changes appear to be more pronounced in the insulin-dependent diabetic subjects. The data support an increase in a broad spectrum of matrix metalloproteinases and immunoglobulins in the recently ruptured plaques, comparable to that seen in non-diabetic subjects. Finally, our analysis suggests an increase in intraplaque neovascularization accompanied by plaque rupture. Future studies that directly compare changes in the transcriptome and cellular composition in specific plaque regions between diabetic and non-diabetic subjects are thus needed to understand the mechanisms leading to atherosclerotic plaque rupture.

## CRediT authorship contribution statement

**Angelle Bradford:** Writing – original draft, Visualization, Validation, Methodology, Investigation, Formal analysis, Data curation. **Tadashi Yoshida:** Resources, Formal analysis, Data curation. **Sergiy Sukhanov:** Resources, Formal analysis, Data curation. **Foster F. Woods:** Investigation, Formal analysis, Data curation. **Patrice Delafontaine:** Writing – review & editing, Supervision, Resources, Project administration, Methodology, Funding acquisition. **Hernan A. Bazan:** Writing – review & editing, Resources, Project administration, Methodology, Investigation, Formal analysis, Conceptualization. **T. Cooper Woods:** Writing – review & editing, Visualization, Validation, Supervision, Software, Resources, Project administration, Methodology, Investigation, Funding acquisition, Formal analysis, Data curation, Conceptualization.

## Author contributions

A. B., H.A.B. and T.C.W. conceived and designed research; A. B., S.S., T.Y., F.F.W. and T.C.W. performed experiments; A. B., H.A.B. and T.C.W. analyzed data; A. B., H.A.B. and T.C.W. interpreted results of experiments; A. B. and T.C.W. prepared figures; A. B. and T.C.W. drafted manuscript; A. B., S.S., T.Y., F.F.W., P.D., H.A.B., and T.C.W. edited and revised manuscript; A. B., S.S., T.Y., F.F.W., P.D., H.A.B., and T.C.W. approved final version of manuscript.

## Financial support

10.13039/100000002National Institutes of Health, 10.13039/100000050National Heart, Lung & Blood Institute, Grant/Award Numbers: R01HL127092 (to TCW) and R01HL070241 (to PD); 10.13039/100000002National Institutes of Health, 10.13039/100000057National Institute of General Medical Sciences, Grant/Award Numbers: P30GM103337 and U54GM104940; 10.13039/100000002National Institutes of Health, 10.13039/100006108National Center for Advancing Translational Science, Grant/Award Numbers: UM1TR004771 (to PD). Eye, Ear, Nose & Throat Foundation, Grant/Award Numbers: EE220703 and EE220803 (to TCW).

## Declaration of competing interest

The authors declare the following financial interests/personal relationships which may be considered as potential competing interests:

T. Cooper Woods reports financial support was provided by National Heart Lung and Blood Institute. Patrice Delafontaine reports financial support was provided by National Heart Lung and Blood Institute. Patrice Delafontaine reports financial support was provided by NIH National Institute of General Medical Sciences. T. Cooper Woods reports financial support was provided by Eye, Ear, Nose and Throat Foundation. Patrice Delafontaine reports financial support was provided by National Center for Advancing Translational Sciences. The other authors declare that they have no known competing financial interests or personal relationships that could have appeared to influence the work reported in this paper.

## References

[1] Carr S., Farb A., Pearce W.H., Virmani R., Yao J.S. (1996). Atherosclerotic plaque rupture in symptomatic carotid artery stenosis. J Vasc Surg.

[2] Milei J., Parodi J.C., Fernandez Alonso G., Barone A., Beigelman R., Ferreira L.M. (1996). Carotid atherosclerosis. Immunocytochemical analysis of the vascular and cellular composition in endarterectomies. Cardiologia.

[3] Bennett M.R., Sinha S., Owens G.K. (2016). Vascular smooth muscle cells in atherosclerosis. Circ Res.

[4] Butcovan D., Mocanu V., Baran D., Ciurescu D., Tinica G. (2016). Assessment of vulnerable and unstable carotid atherosclerotic plaques on endarterectomy specimens. Exp Ther Med.

[5] Galis Z.S., Sukhova G.K., Lark M.W., Libby P. (1994). Increased expression of matrix metalloproteinases and matrix degrading activity in vulnerable regions of human atherosclerotic plaques. J Clin Invest.

[6] Virmani R., Kolodgie F.D., Burke A.P., Finn A.V., Gold H.K., Tulenko T.N. (2005). Atherosclerotic plaque progression and vulnerability to rupture: angiogenesis as a source of intraplaque hemorrhage. Arterioscler Thromb Vasc Biol.

[7] Purushothaman K.R., Purushothaman M., Muntner P., Lento P.A., O’Connor W.N., Sharma S.K. (2011). Inflammation, neovascularization and intra-plaque hemorrhage are associated with increased reparative collagen content: implication for plaque progression in diabetic atherosclerosis. Vasc Med.

[8] Nikolajczyk B.S. (2010). B cells as under-appreciated mediators of non-auto-immune inflammatory disease. Cytokine.

[9] C. American Diabetes Association pProfessional pPractice (2022). 9. Pharmacologic approaches to glycemic treatment: standards of medical care in Diabetes-2022. Diabetes Care.

[10] Pilla S.J., Yeh H.C., Juraschek S.P., Clark J.M., Maruthur N.M. (2018). Predictors of insulin initiation in patients with type 2 diabetes: an analysis of the look AHEAD randomized trial. J Gen Intern Med.

[11] Alfaraj S.A., Vos R.C., Spruit M., Groenwold R.H., Mook-Kanamori D.O. (2025). Sociodemographic and biological determinants of insulin initiation in type 2 diabetes: a cohort study using routinely collected primary care data. The British journal of general practice: the journal of the Royal College of General Practitioners.

[12] Bazan H.A., Brooks A.J., Vongbunyong K., Tee C., Douglas H.F., Klingenberg N.C. (2022). A pro-inflammatory and fibrous cap thinning transcriptome profile accompanies carotid plaque rupture leading to stroke. Sci Rep.

[13] Trapnell C., Roberts A., Goff L., Pertea G., Kim D., Kelley D.R. (2012). Differential gene and transcript expression analysis of RNA-seq experiments with TopHat and cufflinks. Nat Protoc.

[14] Xu G., Deng N., Zhao Z., Judeh T., Flemington E., Zhu D. (2011). SAMMate: a GUI tool for processing short read alignments in SAM/BAM format. Source Code Biol Med.

[15] Robinson J.T., Thorvaldsdottir H., Winckler W., Guttman M., Lander E.S., Getz G. (2011). Integrative genomics viewer. Nat Biotechnol.

[16] Love M.I., Huber W., Anders S. (2014). Moderated estimation of fold change and dispersion for RNA-seq data with DESeq2. Genome Biol.

[17] Soneson C., Love M.I., Robinson M.D. (2015). Differential analyses for RNA-seq: transcript-level estimates improve gene-level inferences. F1000Res.

[18] Liao J., Qian J., Fang Y., Chen Z., Zhuang X., Zhang N. (2022). De novo analysis of bulk RNA-seq data at spatially resolved single-cell resolution. Nat Commun.

[19] Higashi Y., Sukhanov S., Shai S.Y., Danchuk S., Snarski P., Li Z. (2020). Endothelial deficiency of insulin-like growth factor-1 receptor reduces endothelial barrier function and promotes atherosclerosis in Apoe-deficient mice. Am J Physiol Heart Circ Physiol.

[20] Mocci G., Sukhavasi K., Ord T., Bankier S., Singha P., Arasu U.T. (2024). Single-cell gene-regulatory networks of advanced symptomatic atherosclerosis. Circ Res.

[21] Singh P., Sun J., Cavalera M., Al-Sharify D., Matthes F., Barghouth M. (2024). Dysregulation of MMP2-dependent TGF-ss2 activation impairs fibrous cap formation in type 2 diabetes-associated atherosclerosis. Nat Commun.

[22] Bazan H.A., Hatfield S.A., O’Malley C.B., Brooks A.J., Lightell D., Woods T.C. (2015). Acute loss of miR-221 and miR-222 in the atherosclerotic plaque shoulder accompanies plaque rupture. Stroke.

[23] Coleman C.B., Lightell D.J., Moss S.C., Bates M., Parrino P.E., Woods T.C. (2013). Elevation of miR-221 and -222 in the internal mammary arteries of diabetic subjects and normalization with metformin. Mol Cell Endocrinol.

[24] Narayanan S., Vuckovic S., Bergman O., Wirka R., Verdezoto Mosquera J., Chen Q.S. (2025). Atheroma transcriptomics identifies ARNTL as a smooth muscle cell regulator and with clinical and genetic data improves risk stratification. Eur Heart J.

[25] Lightell D.J., Moss S.C., Woods T.C. (2018). Upregulation of miR-221 and -222 in response to increased extracellular signal-regulated kinases 1/2 activity exacerbates neointimal hyperplasia in diabetes mellitus. Atherosclerosis.

[26] Jonas M., Edelman E.R., Groothuis A., Baker A.B., Seifert P., Rogers C. (2005). Vascular neointimal formation and signaling pathway activation in response to stent injury in insulin-resistant and diabetic animals. Circ Res.

[27] Suzuki S., Suzuki S., Sato-Nagaoka Y., Ito C., Takahashi S. (2024). Identification of triciribine as a novel myeloid cell differentiation inducer. PLoS One.

[28] Tian Q., Chen J.H., Ding Y., Wang X.Y., Qiu J.Y., Cao Q. (2024). EGR1 transcriptionally regulates SVEP1 to promote proliferation and migration in human coronary artery smooth muscle cells. Mol Biol Rep.

[29] Watanabe T., Pakala R., Katagiri T., Benedict C.R. (2001). Synergistic effect of urotensin II with serotonin on vascular smooth muscle cell proliferation. J Hypertens.

[30] Chiu C.Z., Wang B.W., Shyu K.G. (2014). Angiotensin II and the JNK pathway mediate urotensin II expression in response to hypoxia in rat cardiomyocytes. J Endocrinol.

[31] McCartan D., Bolger J.C., Fagan A., Byrne C., Hao Y., Qin L. (2012). Global characterization of the SRC-1 transcriptome identifies ADAM22 as an ER-independent mediator of endocrine-resistant breast cancer. Cancer Res.

[32] Lee S.K., Kim H.J., Na S.Y., Kim T.S., Choi H.S., Im S.Y. (1998). Steroid receptor coactivator-1 coactivates activating protein-1-mediated transactivations through interaction with the c-Jun and c-Fos subunits. J Biol Chem.

[33] Zhang S.M., Jiang L., Zhao X., Liu J.F., Liang B., Liu C. (2019). A disintegrin and metalloprotease 22 accelerates neointima formation by activating ERK signaling. Atherosclerosis.

[34] Papaspyridonos M., Smith A., Burnand K.G., Taylor P., Padayachee S., Suckling K.E. (2006). Novel candidate genes in unstable areas of human atherosclerotic plaques. Arterioscler Thromb Vasc Biol.

[35] Müller A., Krämer S.D., Meletta R., Beck K., Selivanova S.V., Rancic Z. (2014). Gene expression levels of matrix metalloproteinases in human atherosclerotic plaques and evaluation of radiolabeled inhibitors as imaging agents for plaque vulnerability. Nucl Med Biol.

[36] Peeters W., Moll F.L., Vink A., van der Spek P.J., de Kleijn D.P., de Vries J.P. (2011). Collagenase matrix metalloproteinase-8 expressed in atherosclerotic carotid plaques is associated with systemic cardiovascular outcome. Eur Heart J.

[37] Molloy K.J., Thompson M.M., Jones J.L., Schwalbe E.C., Bell P.R., Naylor A.R. (2004). Unstable carotid plaques exhibit raised matrix metalloproteinase-8 activity. Circulation.

[38] Pelisek J., Rudelius M., Zepper P., Poppert H., Reeps C., Schuster T. (2009). Multiple biological predictors for vulnerable carotid lesions. Cerebrovasc Dis.

[39] Nagase H., Visse R., Murphy G. (2006). Structure and function of matrix metalloproteinases and TIMPs. Cardiovasc Res.

[40] Visse R., Nagase H. (2003). Matrix metalloproteinases and tissue inhibitors of metalloproteinases: structure, function, and biochemistry. Circ Res.

[41] Liu X.Q., Mao Y., Wang B., Lu X.T., Bai W.W., Sun Y.Y. (2014). Specific matrix metalloproteinases play different roles in intraplaque angiogenesis and plaque instability in rabbits. PLoS One.

[42] Johnson J.L., George S.J., Newby A.C., Jackson C.L. (2005). Divergent effects of matrix metalloproteinases 3, 7, 9, and 12 on atherosclerotic plaque stability in mouse brachiocephalic arteries. Proc Natl Acad Sci USA.

[43] Tay C., Liu Y.H., Kanellakis P., Kallies A., Li Y., Cao A. (2018). Follicular B cells promote atherosclerosis via T cell-mediated differentiation into plasma cells and secreting pathogenic immunoglobulin G. Arterioscler Thromb Vasc Biol.

[44] Tay C., Liu Y.H., Hosseini H., Kanellakis P., Cao A., Peter K. (2016). B-cell-specific depletion of tumour necrosis factor alpha inhibits atherosclerosis development and plaque vulnerability to rupture by reducing cell death and inflammation. Cardiovasc Res.

[45] Silva P., Hernández N., Tapia H., Gaete-Ramírez B., Torres P., Flores T. (2024). Tumor-derived hypoxic small extracellular vesicles promote endothelial cell migration and tube formation via ALS2/Rab5/β-catenin signaling. FASEB J.

[46] Zhang X., Xu R., Wang T., Li J., Sun Y., Cui S. (2024). PTP1B modulates carotid plaque vulnerability in atherosclerosis through Rab5-PDGFRβ-mediated endocytosis disruption and apoptosis. CNS Neurosci Ther.

[47] Taylor A.C., Murfee W.L., Peirce S.M. (2007). EphB4 expression along adult rat microvascular networks: EphB4 is more than a venous specific marker. Microcirculation.

[48] Du W., Huang L., Tang X., Li J., Li X. (2020). Ephrin-A5 is involved in retinal neovascularization in a mouse model of oxygen-induced retinopathy. Biomed Res Int.

[49] Li Z., Paik J.H., Wang Z., Hla T., Wu D. (2005). Role of guanine nucleotide exchange factor P-rex-2b in sphingosine 1-phosphate-induced Rac1 activation and cell migration in endothelial cells. Prostaglandins Other Lipid Mediat.

[50] Lechleider R.J., Ryan J.L., Garrett L., Eng C., Deng C., Wynshaw-Boris A. (2001). Targeted mutagenesis of Smad1 reveals an essential role in chorioallantoic fusion. Dev Biol.

